# A43 MECHANISMS OF UNIQUE COLONIZATION DYNAMICS AND IMMUNE RESPONSES TO MURIBACULACEAE

**DOI:** 10.1093/jcag/gwad061.043

**Published:** 2024-02-14

**Authors:** S Popple, D Pepin, H Ghezzi, C Tropini, L Osborne

**Affiliations:** Microbiology & Immunology, The University of British Columbia, Vancouver, BC, Canada; Microbiology & Immunology, The University of British Columbia, Vancouver, BC, Canada; Microbiology & Immunology, The University of British Columbia, Vancouver, BC, Canada; Microbiology & Immunology, The University of British Columbia, Vancouver, BC, Canada; Microbiology & Immunology, The University of British Columbia, Vancouver, BC, Canada

## Abstract

**Background:**

Commercially available probiotics have a limited ability to alter the gut microbiome and establish within a pre-existing microbial community. Increases in osmolality, a condition prevalent in intestinal diseases, depletes the highly abundant bacterial family Muribaculaceae (Mb) from the gut microbiome in a mouse model of short-term osmotic laxative use. Excitingly, despite microbial community re-equilibration in its absence, Mb supersedes other bacterial colonizers to its original abundance if reintroduced, suggesting unique colonization attributes and a potential privileged immune interaction.

**Aims:**

We aimed to investigate whether and how Mb colonization affects intestinal innate and adaptive immune homeostasis, including the generation of Mb-specific antibodies.

**Methods:**

To examine interactions between Mb and the immune system that allows for efficient colonization, we used the osmotic laxative polyethylene glycol (PEG) to clear Mb and then generated mice with complex microbiomes that either lack Mb completely (Mb naïve) or were introduced to 8 murine-derived Mb strains (Mb+). The same strains were then introduced to Mb naïve mice (primary exposure to the immune system). To model secondary exposure, Mb+ mice were treated with PEG, and Mb re-introduced 3 weeks later. Leukocytes from the small and large intestinal lamina propria and gut-draining mesenteric lymph nodes (mLNs) were profiled over 21 days to characterize colonization and recolonization dynamics.

**Results:**

High-throughput 16SrRNA sequencing showed no significant difference between relative abundance of Mb isolates during colonization or recolonization, indicating that Mb is capable of establishing in mice that have never been colonized with it before, making up to 20% of the relative abundance. Broad immunophenotyping demonstrated robust CD11b^-^CD103^+^ dendritic cell and Germinal Centre B cell responses in the mLNs of mice seeing Mb for the first time, yet no subsequent plasmablast accumulation in the mLN or lamina propria. We also observed significantly increased type two innate lymphoid cells in the small intestine upon primary exposure to Mb. Recolonization elicited no detectable changes in the monitored populations.

**Conclusions:**

The lack of immune reactivity upon reintroduction to Mb suggests Mb may evade immune detection upon repeat exposure and contribute to its robust colonization abilities. Determining mechanisms involved in Mb colonization and its impact on host immune responses could increase our understanding of colonization dynamics and highlights the potential of Mb as a model for microbiota reintroduction in therapeutic applications.

**Funding Agencies:**

Weston Family Foundation

